# “Descriptive Psychology and the Person Concept”

**DOI:** 10.17505/jpor.2025.28097

**Published:** 2025-06-28

**Authors:** Lars-Gunnar Lundh

**Affiliations:** Department of Psychology, Lund University, Sweden

**Keywords:** Concept of person, Deliberate action, Ossorio, Consciousness, Feeling, Empathy, Parametric analysis

*“What makes an individual a person is, paradigmatically, to have mastered the concept of a Person.” (Peter Ossorio)*

The first chapter of Wynn Schwartz book starts with this quote from the American psychologist Peter Ossorio (1926-2007). Ossorio, who worked at the University of Colorado, US, founded a school that is called “descriptive psychology”, and his most important work is *The Behavior of Persons* (Ossorio, 2006). Schwartz is one of Ossorio’s followers.

One of Ossorio’s and Schwartz’s main claims is that the concept of person is not only basic to psychological science – *it is also basic to our psychological functioning as individuals*. We all have an implicit understanding of what it means to be a person among other persons, and without this understanding we would not be able to function in the way we do:

Generally, if we did not understand people, life would be chaotic. We could not cooperate. You could not understand this sentence. Understanding people *is* the central competence involved in being a person in a world of others. (Schwartz, 2019, p. 4)

This implicit understanding of what it means to be one person among others is fundamentally involved in the interpersonal processes that characterize our society and culture. Moreover, some aspects of this understanding – for example, that people make choices for which they are responsible – are explicitly expressed in laws, contracts, and negotiations of many sorts:

persons hold each other more or less responsible for the choices they make. This is an implicit premise of civilization: awake and aware people have a degree of choice and can weigh their reasons for a course of action. To the extent people choose what they want to do, they are accordingly accountable for their behavior. This accountability is made explicit in the law and in negotiations, sworn contracts, and the like. (Schwartz, 2019, p. 24)

As emphasized by Schwartz, it remains for psychological science to make this implicit understanding *fully explicit* in the form of a pre-empirical and pre-theoretical *conceptual framework* for systematic psychological research and psychological practice.

As he describes it, this is not only a *pre-empirical* but also a *pre-theoretical* task, as the purpose is not to formulate empirically testable theories or hypotheses.

Descriptive Psychology is a *preempirical discipline* structured by the logical requirements of the Person Concept and designed to systematically organize this subject matter’s full range of possibilities. This is odd because psychology is usually anchored in theory. Descriptive Psychology is something else entirely, *something formally prior to theory*… This sort of preempirical conceptualization should provide a modifiable, theory-neutral framework for the systematic study of everything that fits the subject matter. (Schwartz, 2019, p. 55)

In other words, it is an important conceptual-analytical task at the basis of psychological science *to make explicit what we already know implicitly* as functioning individual persons. And although the verbal formulations that are produced by this work are not *empirically testable*, they are still possible to *modify and improve* by means of further *conceptual* work. A method used in the descriptive psychological approach of Ossorio and Schwartz is *paradigmatic case formulation*.

Schwartz’ book contains nine chapters, which apply this kind of analysis to various parts of psychology, including intentional behaviour, judgment, personal relationships, and empathy. My focus in this review is on the definition of the concept of person. I will turn first to the method of paradigmatic case formulation, as illustrated by how it has led, by a series of successive approximations, to the development of a definition of what it means to be person. For short, I will refer to this as “the DP definition” of the concept of person (where DP stands for Descriptive Psychology). Then I will raise some critical questions concerning this definition.

## Paradigmatic Case Formulation (PCF)

Ossorio (1981) developed a method of constructing Paradigm Case Formulations (PCFs) to be used when traditional definitions fail to capture the full possible range of a concept. The basic idea is simple: (1) Specify a paradigm case of the category you are interested in (e.g., “families”, “chairs”, or “persons”). (2) Introduce one or more transformations of the case to see if this violates what you consider to be a case of the category in question

Here’s how to proceed. First, do you agree that the following paradigm case is an example of a family? If so, then look at the transformations. Do any of them violate what you consider a family? Can you see how someone might draw the line differently? Can you accept marginal and ambiguous cases as grounds for reasonable disagreement?

By starting with a paradigm case that everyone readily identifies as within their understanding, it should be possible to delete or change features of the paradigm with the consequence that with each change some people might no longer agree that we are still talking about the same thing. But because of the shared paradigm, it should be possible to show where agreement and disagreement lie and where various reasonable judges draw the line. (Schwartz, 2019, p. 22)

Here it is important to see that this is a procedure where the people involved strive for *intersubjective agreement*. The method is assumed to especially helpful “when definitional boundaries are fuzzy, and disagreement easy” (p. 20).

Finding a fully inclusive definition presents a common conceptual dilemma. Consider how difficult it is to exactly define what is meant by the word “family” or the word “chair” if we wish to achieve agreement on all possible examples of families and chairs… What elements must be present and what can we change, add, or leave out and still honor what different reasonable people call a family? (Schwartz, 2019, p. 20)

These difficulties clearly apply also to the concept of person. And as described by Schwartz, the definition of an individual person within Ossorio’s descriptive psychology “has under-gone a history of refinements” (p. 22).

## The Development of the DP Definition of Persons

In the 1970s, Ossorio suggested that persons could be defined as

individuals whose history is represented by a series of intentional actions.

Interestingly, this definition refers to the individual’s *life history*. As Ossorio (2006) puts it, the “appropriate size of the unit for conceptualizing a person is not a behavior but a life history” (p. 384). This suggests a holistic perspective, where individual behaviors and experience are seen in terms of their role in the person’s life patterns. Although it is a basic characteristic of persons that they engage in deliberate action, these actions are part of larger life-historical patterns. A central role in these patterns is played by the individual’s personal *values*, or more precisely their specific “motivational hierarchy of intrinsic core values” (Schwartz, 2019, p. 43), which give a person’s life its direction by making certain things in life more significant than others. Importantly, these values reveal themselves in the person’s actions, more than in their verbal statements about their values: “The values revealed may not be the values a person claims they hold most dear. Actions speak loudest.” (Schwartz, 2019, p. 43)

This definition, however, proved to be overinclusive as it was found to include animals of other species who also show intentional behaviour. Because we don’t commonly agree to see animals of other species as *persons*, Ossorio suggested that “intentional” should be changed to “deliberate”.

When the dust settled the Descriptive community finally accepted “a person is an individual whose history is, paradigmatically, a history of deliberate action.” (Schwartz, 2019, p. 22)

The significance of this reformulation is that deliberate action is a special case of intentional behaviour, where the individual acts not only for the purpose of achieving a certain goal, but *chooses* to act in this way after having *deliberated* about other *alternative* actions.

Deliberate Action is… a variation of intentional action, and involves an awareness or cognizance of a choice. Deliberate actions are a demonstration of personal autonomy insofar as autonomy is linked to the ability to make choices. (Schwartz, 2019, p. 24)

In other words, *deliberation* and *choice* are seen as capacities that specifically characterize persons. This is clearly in accordance with the common practice of holding persons accountable and responsible for their deliberate actions. The reformulation from “intentional” to “deliberate”, however, was not sufficient. What was added in the final DP definition of the concept of person is that a person doesn’t only have a *history* that involves deliberate action, but that these activities form recurring patterns over time:

a person is an individual whose history is, paradigmatically, a history of Deliberate Action in a dramaturgical pattern.

As Schwartz comments, it might be even better to speak about dramaturgical pattern*s* in plural, “since people have a multiplicity of personally significant concerns, entertained in their various ways” (Schwartz 2019, p. 29).

Why then are these patterns called “dramaturgical”? Why isn’t it sufficient to speak about *recurring* patterns? The answer seems to be that the person is seen in analogy with an actor. To quote some of Schwartz’ (2019) formulations:

a person’s life is akin to the drama of an improvisational play (p. 41)

Human lives are intrinsically and fundamentally dramatic in form” (p. 42).

Other researchers with an interest in people’s life history have advocated methods such as narrative analysis. According to Sarbin (1986), for example, who coined the term “narrative psychology”, *stories* are useful to understand human conduct. And in McAdams’ (2001) life story model of identity, people living in modern societies are seen to “provide their lives with unity and purpose by constructing internalized and evolving narratives of the self” (p. 100). The concept of “dramaturgical” patterns, however, can be said to be broader (or go “deeper”) than the concept of narratives, in the sense that it refers to the patterns in people’s lives *that they try to capture be means of stories, narratives*.

Why drama rather than narrative?… Dramaturgical shares key features but is more inclusive than the compatible idea of narrative. As a form of verbal behavior, we voice and write narratives, that when nuanced enough, capture the dramas we live. (Schwartz, 2019, p. 29)

People’s narratives are *about* the dramas of their lives, but narratives may well fail to capture the *real* dramas.

## What About Experience?

My main critique of the DP definition is that it fails to take people’s *experiences* into account. It can be argued that the having of experiences belongs to the most basic characteristics of persons. In his work on psychology from “the personalistic standpoint” Wilhelm Stern (1935), for example, a central role was given to the individual as “a person with the capacity for experience” (p. 84).

The DP definition has a strong focus on patterns of *action* or *behaviour*. The person is seen primarily as an *actor*. As Schwartz puts it, “[a]ll of what we can attribute to an individual can be represented by that individual’s behavior” (p. 2-3). The reason for this focus on behaviour is clearly stated:

Descriptive Psychology’s Person Concept provides a foundation for behavioral science so naturally the focus is the behavior of persons. (Schwartz, 2019, p. 7)

But is psychology only a science of *behaviour*? And can the concept of person really be adequately defined without referring to people’s capacity for experience?

Among other things, the strong focus on *behaviour* is seen also in the fact that Schwartz is willing to attribute person status even to robots:

“A Robot is an individual who is a Person and has a non-biological embodiment.” (p. 23)

Behavioural patterns (in contrast to experiences) are possible to simulate. If a robot is programmed to behave in a way that fulfills Ossorio/Schwartz’ behavioural criteria for personhood, it follows (according to their DP definition) that the robot is a person – *whether it has any feelings or other experiences*. As Schwartz puts it,

The analysis of Deliberate Action might be of use in constructing an artificial intelligence able to act deliberately” (p. 60).

But does this mean that the AI is a person? And if not, what about the DP definition of the person concept?

Robots may well possess *intelligence*, but intelligence is not sufficient for being a person. Persons are not only behaving creatures with a cognitive capacity, but they also have *feelings*, and these feelings are *expressed* in movements. Can we really speak about *persons* in the absence of feelings and experiences?

What is at stake here is not only emotional experiences, but also more basic bodily feelings of the kind that are discussed by Langer (1967) in terms of “felt life”, and by other writers (e.g., Vendrell Ferran, 2021) in terms of feelings of vitality. According to Langer, such feelings form an intricate dynamic pattern of tremendous complexity, where terms such as “excitement,” “calm,” “joy,” “sorrow,” “anxiety,” “love,” and “hate” only represent crude designations. What eludes the power of language, as she puts it, is

the way feelings, emotions, and all other subjective experiences come and go—their rise and growth, their intricate synthesis that gives our inner life unity and personal identity. (Langer 1957, p. 7)

Vendrell Ferran (2021) describes “feelings of vitality” in a similar way as

a broad range of experiences in which we feel the powers of life, its increments and decrements, its ups and downs. Paradigmatic examples are feeling energetic or dispirited, vigorous or weary, full of life or exhausted. In extreme fatigue, in bodily pain, in illness, or at the end of life, we can feel how life fades away from us; in excitement, in happiness, and in feeling renewed, we feel life pulsing through us. We are also able to empathize and feel with others’ feelings of vitality: we perceive the feebleness, the tiredness, the brightness and the vigor in other living beings. (Vendrell Ferran, 2021, p. 116)

Importantly, these bodily feelings are not *about* the body as an object, which could possibly be simulated by a machine. What is involved here is rather *how* the body feels from a first-person perspective. In other words, these are experiences *at the very core of being a person*.

Moreover, these kinds of feelings are typically *expressed* in a way that makes them open to others’ perception. In a classical experiment, Heider and Simmel (1944) showed that when simple animated geometric shapes were moving around on a screen, seemingly on their own accord (i.e., without being pushed into action by some external cause), observers tended to perceive them as personal characters with emotions, intentions, and other experiences. What this shows is that certain patterns of *movement* tend to be directly perceived as *expressive* of feelings and intentions. Even though we do not in any way tend to confuse these geometric shapes with real persons, we still have an almost irresistible tendency to *directly perceive* these movements as expressive of personal feelings and intentions.

In other words, these perceptions are intrinsically connected with *our intuitive understanding of what it means to be a person*. And yet, these phenomena are completely missing in the DP definition of persons.

This bias for behaviour and against experiences is seen also Schwartz’ discussion of the nature of emotions:

Emotions are recognized by what a person does emotionally, not by how their body feels. Actions speak, ‘feelings’ in themselves don’t. (Schwartz, 2019, p. 138).

Here he refers to Wittgenstein’s (1953) argument against the possibility of a private language, as illustrated by the following thought experiment by Bergner (2003):

His thought experiment begins with wanting to buy paint that matches a particular color: Take the paint chip you want to match, go off to the local hardware store, and ask a clerk what it’s called. The clerk consults a chart, says it’s called “autumn gold,” sells you a can, and reassures you that whenever you need more, just ask for “autumn gold.”… Bergner explains that this easily shared and verifiable name, “autumngold,” cannot happen with sensations or feelings. “Suppose, for example, that I have a novel feeling or sensation. I decide to name this sensation ‘arby’. Now clearly there is no way that I can exhibit this feeling, as I did my paint chip, to other persons and have them report, ‘Oh, yes, I have just that same feeling.’ There is no way for them to observe my feeling and thus determine if they experience one that matches mine.”

Words for emotions, the argument goes, “cannot be based on sensations, feelings, affects, or anything else that is not inherently sharable and plausibly verified as the same” (Schwartz, 2019, p. 139). What is missing from this argument is the fact that feelings are *expressed non-verbally* in a kind of “body language” that is *intersubjectively available*. And body language is not a private language.

Wittgenstein’s (1953) argument against the possibility of a private language is *not* an argument against the intersubjective availability of feelings. Consider, for example, his following suggestion:

Just try – in a real case – to doubt someone else’s fear or pain. (Wittgenstein, 1953, § 303).

People who experience fear or pain typically show bodily expressions that we immediately tend to perceive as fear and pain, respectively. What Wittgenstein’s argues against is the mistaken tendency to see words for sensations and feelings as referring to a kind of *objects*. As he exclaims in response to the critique that for him the sensation seems to be “a nothing”:

Not at all. It is not a *something*, but not a *nothing* either! (Wittgenstein, 1953, § 304).

What causes confusion here seems to be the grammatical mistake of taking sensations and feelings to be a kind of *things* or *objects* for our verbal thinking. Sensations and feelings are much closer to us than that – which is seen also in the fact that there is no way of being mistaken about feeling pain. We may be wrong about many things, but to feel pain is to *be* in pain. Feelings pertain to our very *being* as individual persons.

It should be emphasized that what is most important here are still the feelings, not the expressions. The theoretical possibility cannot be excluded that it might be possible to simulate bodily expressions in a robot in such a way that we might tend to perceive these as expressions of real feelings (cf. the study by Heider & Simmel [1944] as referred to above).

Feelings are conscious experiences. The neuroscientist Anil Seth (2021) has formulated an interesting thought experiment, which shows the immense importance of conscious experiences to us as individual persons. The thought experiment goes as follows: Suppose you are offered the deal of having your brain replaced “with a machine that is its equal in every way, so that from the outside, nobody could tell the difference” (p. 3). Suppose further that one of the many advantages in favour of this deal is that this new machine is immune to decay and may perhaps even allow you to live forever. At the same time, a crux is that the scientist who provides the offer *cannot* guarantee that you will have any conscious experiences at all, if you should take up this offer. How would you respond? Seth’s guess is that:

I suspect you wouldn’t take the deal. Without consciousness, it may hardly matter whether you live for another five years or another five hundred. In all that time *there would be nothing it would be like to be you*. (Seth, 2021, p. 3-4).

As Seth (2021) summarizes it:

For each of us, our conscious experience is all there is. Without it there is nothing at all: no world, no self, no interior and no exterior. (p. 3).

Or, in other words, being a person is not only a matter of intentionality (whether it is defined in terms of intentional action or intentional objects of thought), but it also essentially involves conscious *experiences*.

## Empathy and Perspective-Taking

In view of my reservations against the DP definition of the concept of person, I expected that to find a similar bias for the cognitive aspects of empathy also in Schwartz’ chapter on empathy. My way of approaching this chapter was in terms of an understanding of empathy as involving two different aspects of: *felt empathy* and *perspective taking*. As defined elsewhere,

felt empathy means to immerse oneself into *how the other person may feel “from within”*, whereas perspective-taking means to immerse oneself into *what the other person’s perspective on the world (including their own body as an object) may be like*. Again, it may be argued that optimal empathy is to be seen when these two aspects (felt empathy and perspective taking) go together. (Lundh & Foster, 2025)

In terms of this differentiation, I expected that Schwartz’ chapter on empathy would have a bias for perspective-taking, with little attention to felt empathy. These expectations were at least partly confirmed.

In the beginning of the chapter, however, Schwartz quotes Carl Rogers and Heinz Kohut, who both have a balanced definition of empathic understanding, which includes both perspective-taking and felt empathy. Rogers (1975) is quoted as stating that empathy involves “entering the private perceptual world of the other,” (i.e., perspective-taking) and “being sensitive, moment to moment, to the changing felt meanings which flow in this other person” (i.e., felt empathy). And Kohut (1984) is quoted as saying that empathy is “the capacity to think and feel oneself into the life of the other person” (thereby referring to perspective-taking and felt empathy in the same sentence).

In the remainder of Schwartz’ chapter, however, there is an almost complete focus on perspective-taking, and little or no mention of felt empathy. For example, on p. 211, he states that “Caring and accurate attention to perspective, explicitly stated or otherwise shown, is empathy.” And on p. 213, it is stated that

Empathic action communicates an accurate understanding of the significance of a person’s behaviors and circumstances in a way they can tolerate… This requires appreciating their current perspective by understanding their active motivations; their relevant knowledge of themselves and their circumstances; their relevant know how; and the significance to them, of their performance.

Again, empathy is defined in terms of taking the other’s perspective, with no mention of what the person might feel. There is, however, at least one implicit reference to feelings in the clause that empathic understanding should be communicated in a way that the other person is able to *tolerate*.

To summarize, the absence of reference to feelings and experiences in the DP definition of the concept of person is mirrored in Schwartz’ discussion of empathy. This does not mean that his chapter on empathy is not worth reading – in fact, I think it has many qualities. My main point, again, is simply that there seems to be something basic missing from the DP definition of what it means to be a person.

## Parametric Analysis

Another descriptive-psychological methodological tool outlined by Ossorio and Schwartz is *parametric analysis*. A parameter is defined as “a necessary and independent” dimension of the subject matter. A simple example is colour, which can be analyzed in terms of three parameters: hue, saturation, and brightness. Parametric analysis is also applied to psychological phenomena, such as intentional action. The parameters need not be quantitative as in the case of colour (where the values of hue, saturation, and brightness can be given as numbers), although they must show some kind of variation within a certain dimension:

The only restriction is that all the values of a given parameter are of the same kind. (Ossorio, 2006, as quoted by Schwartz, 2019, p. 65)

Applying parametric analysis to intentional action, Schwartz (2019, p. 66) describes eight parameters as necessary and independent dimensions for the identification and differentiation of intentional actions:

**Intentional Action (IA) = <I, W, K, KH, P, A, S, PC>,** where:

– **I** is the **Identity** of the actor (e.g., as given by the actor’s name),
– **W** is what the actor **Wants** to accomplish in the situation,
– **K** is what the actor **Knows** that is relevant to is wanted
– **KH** is what the actor **Knows How** to do, given what is wanted and what is known
– **P** is the **Performance** of the action in real time
– **A** is the **Achievement** or outcome of the behavior
– **S** is the **Significance** of the action for the actor
– **PC** is the **Personal Characteristics** expressed by the action (e.g., people vary in their capacities and dispositions)

Again, there is no mention of feelings or experiences. For example, it might be added that a person also has an *experience* of how it *feels* to carry out a given action – and that this can be *expressed* in the actor’s physical *movements* in a way that is fully observable for others. For example, an action can be performed either uncertainly, hesitantly, or in a very determined manner, and this may have considerable impact on the outcome of the action. (And this cannot be subsumed under the parameter of Personal Characteristics, which refers to skills and traits, rather than momentary feelings.)

A sympathetic feature of this kind of approach, however, is that Schwartz clearly states that the IA formulation, and all such formulations (or formulas), are open to modification based on further analysis:

The IA formulation should provide common ground and span all of behavioral science, but if found inadequate or incomplete, it can be refined, expanded, or modified. This flexibility is required for an open-to-possibility, theory-neutral subject domain. (Schwartz, 2019, p. 66)

## Conclusion

One of the things I find most interesting in Ossorio’s and Schwartz’s work is their claim that the concept of person is not only basic to psychological *science*, but also to *our functioning as persons* – we all have an implicit understanding of what it means to be a person among others, without which we would not be able to function in the way we do. Still, I find their analysis of this implicit understanding of the person concept rather one-sided, with an overexaggerated focus on intentional action and deliberation and a relative disregard for the role of feelings and experiences.

Hopefully, their descriptive-psychological approach can leave room for further developments in making the implicit explicit. Schwartz’ book breathes an openness to modification and expansion of their descriptive-psychological analyses:

Building a framework where all the facts can be located requires considerable care and an openness that allows modification and expansion. (Schwartz, 2019, p. 6)

It is also interesting to speculate about the potential of such an analysis, and what the implications are of an expanded and deepened self-understanding of what it means to be a person among other persons. As Schwartz puts it, we humans are “a work in progress”.

Our descendants, if we don’t blow ourselves to extinction, may look back and find our perspective underdeveloped. We’re a work in progress. (Schwartz, 2019, p. 51)

*Lars-Gunnar Lundh*


## Open access

This article is distributed under the terms of the Creative Commons Attribution 4.0 International License (http://creativecommons.org/licenses/by/4.0/), which permits unrestricted use, distribution, and reproduction in any medium, provided you give appropriate credit to the original author(s) and the source, provide a link to the Creative Commons license, and indicate if changes were made.
